# A Novel Bio-Psychosocial-Behavioral Treatment Model in Schizophrenia

**DOI:** 10.3390/ijms18040734

**Published:** 2017-03-30

**Authors:** Yong-Ku Kim, Joonho Choi, Seon-Cheol Park

**Affiliations:** 1 Department of Psychiatry, Korea University College of Medicine, Seoul 02841, Korea; yongku@korea.edu; 2 Department of Psychiatry, Hanyang University Guri Hospital, Guri 11923, Korea; jchoi@hanyang.ac.kr; 3 Department of Psychiatry, Inje University College of Medicine and Haeundae Paik Hospital, Busan 48108, Korea

**Keywords:** schizophrenia, bio-psychosocial-behavioral, model, neurodevelopment, neural circuitry, cognitive

## Abstract

Despite the substantial burden of illness in schizophrenia, there has been a discrepancy between the beneficial effects of an increased use of antipsychotic medications and achieving limited recovery or remission. Because the focus of the most common antipsychotic medications is on dopamine, which is associated with positive symptoms, there is an unmet need for patients with negative symptoms. Since cognitive and negative symptoms rather than positive symptoms are more closely associated with psychosocial impairments in patients with schizophrenia, the non-dopaminergic systems including glutamate and γ-aminobutyric acid (GABA) of the prefrontal cortex should be of concern as well. The balance of excitation and inhibition has been associated with epigenetic modifications and thus can be analyzed in terms of a neurodevelopmental and neural circuitry perspective. Hence, a novel bio-psychosocial-behavioral model for the treatment of schizophrenia is needed to account for the non-dopaminergic systems involved in schizophrenia, rather than dopaminergic mechanisms. This model can be understood from the viewpoint of neurodevelopment and neural circuitry and should include the staging care, personalized care, preventive care, reducing the cognitive deficits, and reducing stigma. Thomas R. Insel proposed this as a goal for schizophrenia treatment to be achieved by 2030.

## 1. Introduction

Schizophrenia afflicts 0.5–1% of the world’s population and is clinically characterized by impaired social and occupational functioning associated with misattributions or delusions, hallucinations, cognitive deficits, thought disorder, negative symptoms, mood changes, and movement disorder. Most patients with schizophrenia begin to present paranoid delusions and auditory hallucinations in late adolescence or in early adulthood [1,2]. In the global burden of mental disorders, schizophrenia is ranked third following unipolar depressive disorders and alcohol-use disorders with 14.4 million disability-adjusted life years (DALYs) by the Grand Challenges in Global Mental Health Initiative [3]. Thus, the supply of effective and better treatments should be improved, and more effective and affordable community-based care and rehabilitation techniques should be provided in order to reduce costs and burden of severe mental illnesses including schizophrenia [4,5].

Despite this being a “golden age of psychopharmacology” [6,7], there is a discrepancy between the beneficial effects of psychotropic medications and the lack of improvement for people with schizophrenia [6,7]. Despite the revolutionary advances in genomics and neuroscience in the realm of clinical psychiatry, the diagnostic methods for schizophrenia remain relatively unchanged [8,9,10]. Diagnosing schizophrenia not by the underlying biological underpinnings but by consensus on symptoms may be associated with significant heterogeneity in the clinical course and diversity in treatment responses to antipsychotics [6]. Instead, clinical targets should be changed from symptoms to biomarkers or endophenotypes and clinical treatments should be more sophisticated and individualized. This would be in accordance with our improving understanding of schizophrenia, from a disease caused by a chemical imbalance to one caused by dysfunctional circuitry [6,11]. In terms of deconstructing the Kraepelinian dualism of psychoses, a combination of categorical and dimensional representations of psychopathology has been proposed to present the symptoms of affective psychosis and non-affective psychosis [12]. Interestingly, a combination of categorical and dimensional representations is partly reflected by the Clinician-Rated Dimensions of Psychosis Symptom Severity [13,14], not the diagnostic criteria for schizophrenia in Diagnostic and Statistical Manual of Mental Disorders, 5th edition (DSM-5). Thus, the next-generation treatment model for schizophrenia has been proposed to overcome the problems and obstacles in the realm of clinical psychiatry that marked the period between 1980 and 2000 [6].

Hence, translating scientific advances into public health impacts is essential in terms of defining the pathogenesis of disorders from genes to behaviors, mapping the trajectory of illness to determine when, where, and how to intervene to preempt disability, and developing new interventions based on a personalized approach to the diverse needs and circumstances of patients with mental disorders [15]. Additionally, in terms of accelerating the integration of psychiatry with the rest of medicine, the discipline of so-called clinical neuroscience has become a more important theoretical background for psychiatry than traditional models of psychosis [8,9,10]. Thus, the aim of our paper is to present the necessity of a novel bio-psychosocial-behavioral treatment model in accordance with new molecular targets, new clinical targets, and new use of current treatments.

## 2. Unmet Needs in the Current Antipsychotic Medications for Schizophrenia

Although detecting disease-specific medications indicated for diverse symptom domains are ideal for antipsychotic treatments, current antipsychotic medications are more effective for treating positive symptoms than treating negative symptoms, which can include cognitive deficits [16,17]. Thus, the heavy reliance on dopamine D2 antagonists, the dependence on serendipity, the failure to critically examine alleged “major advances”, and the inability to fully apply the emerging picture of schizophrenia neurobiology has been the common theme of schizophrenia treatment for the past two decades. Hence, schizophrenia has been regarded as a “dopamine disorder” and the research has tended to seek a “pharmacological magic bullet” [18,19]. In a similar vein, dopamine overdrive in schizophrenia has been modeled as a dopamine supersensitivity psychotic reaction. It has been modeled as a process of an excessive pruning of presynaptic neurons in the thalamus, an increase in endogenous dopamine release, a decrease in thalamic presynaptic dopamine D2 receptors, increased dopamine D2high receptors in the hippocampus, frontal cortex, and substantia nigra and an increase in the proportion of D2D2 dimers and D1D2 dimers in the striatum. Hence, the focus of therapeutic interventions in schizophrenia has been preventing dopamine overdrive [20,21,22,23,24]. However, the etiology of schizophrenia is actually more complex than these models would allow, being far more complex and multi-causal than the dopamine overdrive theory suggests [18,19]. Hence, the arbitrary diagnosis of schizophrenia with little biological validity, few acceptable animal models, a lack of actionable biomarkers, and other factors are the obstacles for detecting the novel therapeutic targets for schizophrenia [25]. Moreover, the negative symptoms and cognitive deficits rather than positive symptoms are a major reason for the social and occupational dysfunctions in patients with schizophrenia and are not effectively treated with current antipsychotic medications [16,26,27,28].

### 2.1. Treatments for Negative and Cognitive Symptoms

To treat negative symptoms, glycine transporter inhibitors (e.g., bitopertin), metabotropic M_2_/M_3_ agonists (e.g., pomeglumetad methionil), nicotinic and muscarinic cholinergic agents, antibiotics (e.g., minocycline), hormones (e.g., oxytocin, the estrogen receptor modulator raloxifene and pregnenolone), and targeting glutamate receptors have been evaluated as novel targets. However, the results were inconclusive [29,30,31,32,33,34,35,36]. Additionally, glycinergic agents, glycine transport inhibitors, α_7_ nicotinic and M_1_ muscarinic agonists and allosteric modulators, phophodiesterase (PDE) inhibitors and kynurenine aminotransferase (KAT II) inhibitors have been regarded as the novel approaches for cognitive dysfunctions. Unfortunately, the results of these tests have so far proven inconclusive as well [16,27,28,29,37,38].

### 2.2. Targeting Non-Dopaminergic Mechanisms

Non-dopaminergic mechanisms including serotonin, glutamate, γ amino-butyric acid (GABA), acetylcholine, inflammation and oxidative stress have been proposed as the promising therapeutic targets for schizophrenia [25]. First, in terms of targets of serotonin, whereas agents with D2/5-HT_2A_ antagonism and/or 5-HT_2A_ partial agonism are currently used, agents targeting 5-HT_1A_ activity have been evaluated for the treatment of negative and cognitive symptoms with better tolerability. In addition, 5-HT_2C_ agonists and 5-HT_6_ antagonists have been proposed as clinically useful agents for the early or prodromal stage of schizophrenia, and 5-HT_3_ antagonists have been evaluated as adjunctive treatments for negative and cognitive symptoms. However, lurasidone, which has a potent 5-HT_7_ antagonism, has no clinical implications for treating negative and cognitive symptoms [16,39]. 

Second, in terms of glutamate transmission, promising therapeutic agents include glycinergic agents (e.g., serine and cycloserine) and glycine transport inhibitors, which indirectly stimulate the NMDA receptor. In addition, metabotropic 2 and 3 (mGlu2/3) receptor agonists which attenuate excessive glutamate release have clinical implications and are promising as well. However, agents with AMPA receptor activity have inconclusive therapeutic benefit [29,40,41].

Third, in terms of GABA receptor allosteric modulators, selective GABA_A_ agonists and GABA_B_ antagonists and allosteric modulators at GABA_A_ receptor subtypes (α1, 2, 3 and 5) are also promising therapeutic agents for schizophrenia [25]; Fourth, in terms of neuropeptides, cholecystokinin (CCK) receptor agonists, cannabinoid 1 (CB1) receptor antagonists and neurokinin 3 (NK3) receptor antagonists have been proposed as the potential antipsychotic strategies [42,43,44]; Fifth, since PDE contributes to degradation of the intracellular signaling pathways including cyclic AMP and cyclic GMP which have been associated with functions of dopamine and other neurotransmitters, PDE inhibitors have been proposed as potential therapeutic strategies for treating positive, negative and cognitive symptoms [37]; Sixth, since the relationship between elevated levels of kynurenic acid and declines in cognitive function in schizophrenia have been presented, KAT II inhibitors have been proposed as another strategy to treating cognitive symptoms [38]; Seventh, because of a possible relationship between inflammation and schizophrenia, anti-inflammatory agents have been evaluated in treating the early stage of schizophrenia [45,46]; Lastly, anti-oxidants have been evaluated for their ability to enhance neuroprotection and as an adjunctive strategy. 

Recently, a glutathione (GSH) deficit has been associated with alterations in prepulse inhibition, reactivity to stress, NMDA receptor function, GABA receptor function, dopamine transmission and neural synchrony in a mouse model of schizophrenia [25]. *N*-acetyl cysteine increases the levels of GSH and has accordingly been suggested as another potential strategy for treating schizophrenia in a recent systematic review [47].

### 2.3. Multiple Interlocking Models Integrating Dopaminergic and Non-Dopaminergic Mechanisms

In the pathogenesis of schizophrenia, a broad range of genetic, environmental risk factors and their interactions can alter many distinct neurotransmitter circuits, leading to the characteristic cognitive, behavioral and social dysfunctions of the disease. In a model of interlocking nodes with a central hub, representing oxidative balance and the immune system as inputs to the glutamatergic system, imbalance in any of these nodes can affect the entire system [25,48].

## 3. Schizophrenia from the Viewpoint of a Neural Circuitry

The cognitive deficits (e.g., impaired working memory) and negative symptoms (e.g., reduced capacity to recognize spoken emotional tone) of schizophrenia are part of the disease process of schizophrenia. Thus, the dorsolateral prefrontal cortex (DLPFC) and primary auditory cortex are associated with cognitive and negative symptoms, respectively, in schizophrenia [19,49].

### 3.1. Alterations in the Circuitry of Dorsolateral Prefrontal Cortex

Impairment in the working memory of patients with schizophrenia has been consistently characterized by disturbances in the manipulation of stored information and the maintenance of goal representations, which coincide with alterations in the DLPFC circuitry [50,51]. The altered patterns in the DLPFC circuitry for patients with schizophrenia cannot be simply explained, since those with schizophrenia have presented the increased DLPFC activation with tasks requiring low levels of working memory and decreased DLPFC activation with tasks requiring high levels of working memory [52]. The alterations in the DLPFC have been implicated with several components for pyramidal neurons comprising about 75% of cortical neurons and utilizing glutamate, interneurons comprising about 25% of cortical neurons and utilizing GABA and axons from neurons in the thalamus and from dopamine-containing neurons in mesencephalon innervating targets in the DLPFC [19].

The neurodevelopmental model has proposed that abnormality in the normal synaptic pruning and disturbances in the mechanisms of adolescence-related synaptic elimination contribute to excessive synaptic pruning and decreased spine number in patients with schizophrenia. In terms of the defects in pyramidal neurons, alterations in the DLPFC in patients with schizophrenia have been associated with reduced excitatory inputs to the DLPFC deep layer 3 pyramidal neurons, mediated by the actions of AMPA and NMDA receptors on dendritic spines of pyramidal neurons [53]. However, whether there are alterations in mRNA or protein levels of NMDA receptor subunits in the DLPFC remains unknown. Findings from the postmortem studies for patients with schizophrenia have been inconclusive [54,55,56].

Since postmortem studies for patients with schizophrenia revealed reduced levels of mRNA encoding the 67-kDa isoform of glutamic acid decarboxylase (GAD_67_) [57], decreased levels of GABA have been proposed as an improper neurotransmission pattern in the DLPFC. Despite the unchanged number of calcium-binding protein parvalbumin (PVALB) neurons, the reduced expressions of PVALB mRNA have been presented in patients with schizophrenia [58,59]. Reduced levels of GABA transporter 1 (GAT1) reportedly are found in the axon terminals of the chandelier neurons in PVALB-containing interneurons among patients with schizophrenia [60]. Thus, reduced levels of mRNA encoding GAD_67_ in the DLPFC interneurons may be a primary component of disease process and might contribute to pre-and post-synaptic changes including the reduced expressions of PVALB and GAT1 and elevated expression of GABA_A_ receptor α_2_ subunits in patients with schizophrenia [61,62].

Interestingly, inputs from dopamine neurons in the ventral mesencephalon can modulate the activities of pyramidal neurons and GABAergic interneurons. The decreased dopamine signaling via the reduced expression levels of markers of dopamine axons, such as tyrosine hydroxylase, and dopamine transporter, reduced levels of glutamate-mediated excitation to midbrain dopamine and reduced availability of extracellular dopamine in the DLPLC may be associated with working memory impairments in patients with schizophrenia [63,64,65,66].

Overall, these findings suggest the following network model of schizophrenia: hypofunction of NMDA receptors located on PVALB-containing interneurons, reduced expression of GAD_67_, decreased GABA production, disinhibition of pyramidal neurons and excess levels of glutamate at non-NMDA receptors [67]. 

### 3.2. Alterations in the Circuitry of Auditory Cortex

Reduced gray matter volume and an accelerated loss rate of gray matter in Heschl’s gyrus have been observed in patients with schizophrenia. Interesting, an electroencephalogram (EEG) wave known as the mismatch negativity (MMN) is related to the magnitude of progressive reductions in gray matter of Hesch’s gyrus in the early stage of schizophrenia [68]. The MMN in patients with schizophrenia can be mimicked by infusion of NMDA receptor antagonists into the auditory cortex of animals [69]. 

### 3.3. Alterations in the Visual Circuitry

It is known that the morphological and functional anomalies in almost the entire visual tract, from the retina to the cortex, occur in schizophrenia. It has been proposed that these anomalies are due to defects in the neurodevelopmental processes involving genes, neurotrophic factors (e.g., retinoic acid), environment (e.g., alcohol, infection), and/or a combination of these factors. Eye tracking dysfunction (ETD) has been proposed as an endophenotype associated with genetic and environmental factors from the neurodevelopmental perspective in schizophrenia. It has been suggested that the defects in proteins, genes, and neurotrophic expressions during the first four weeks of the embryonic period may be associated with ETD-related anatomical and functional abnormalities. Moreover, retinoic acid has been regarded as a candidate for regulation of the retinal development, since it plays a significant role in the early stages of embryonic development. Hence, the specific vulnerability to perturbation of the retinoic acid receptors rather than other brain areas has been considered to be significantly involved in defects from the neurodevelopmental perspective of schizophrenia. In conclusion, the visual anomalies in schizophrenia might contribute to alterations in the visual circuitry associated with neurodevelopmental defects during the embryonic period. Herein, the visual circuitry has been proposed as a research tool for biological mechanisms of information processing deficits in schizophrenia [70,71].

### 3.4. Novel Therapeutics from the Viewpoint of a Neural Circuitry

Hence, it has been expected that the new types of therapeutic targets beyond manipulating neurotransmitter systems would be identified and validated with analyzing the pathological neural circuits in the etiology and pathogenesis of schizophrenia. One potentially promising therapeutic target are spine-specific kinases, since they regulate spine size, number and functions [72,73].

## 4. Reconceptualizing Schizophrenia from a Neurodevelopmental Perspective

Maternal stress during the first trimester of pregnancy is associated with elevated risk of schizophrenia in male offspring. Moreover, stress is particularly harmful if presented during the phase of development when neural circuits, such as the projection from the hippocampus to the DLPFC, are being formed [74,75]. Thus, events during prenatal and early life may influence the development of schizophrenia. Associations between in utero exposures to prenatal risk factors (infections, hypoxia and starvation) and clinical features of offspring with schizophrenia (disturbances in executive function, working memory and verbal memory and structural brain abnormalities) are observed in large birth cohort studies with clinical, neurocognitive and neuroimaging measures [76,77,78,79,80,81,82]. Prenatal infection and enlargement of the cavum septum pellucidum/diminished intracranial volume is also linked to schizophrenia [83].

Thus, an integrative model of genetic, environmental and epigenetic factors has been suggested in the etiology and pathogenesis of schizophrenia. It has been suggested the existence of allele-specific epigenetic differences in genes associated with psychosis constitute a further element in the dynamic interplay between the environment, genome and epigenome [84]. The influences of mild isolation stress during adolescence in rodents on behavioral profiles and DNA methylation of the gene encoding tyrosine hydroxylase in mesocortical dopaminergic neurons, when combined with a relevant genetic risk including a genetic variant of DISC-1 [85], can cause a schizophrenia-like phenotype. The integration of epigenetic effects into bio-psychosocial-behavioral models of schizophrenia is expected to provide novel insights to the biological underpinnings of the onset and course of schizophrenia [84]. Schizophrenia has been neurodevelopmentally modeled as follows: reduced elaboration of inhibitory pathways and excessive pruning of excitatory pathways in children that develop schizophrenia leads to alterations in the excitatory-inhibitory balance in the prefrontal cortex and reduced myelination. This disorder in synaptic connectivity is directly responsible for the symptoms of schizophrenia, particularly negative symptoms. Identifying what the prodromal neurodevelopmental changes are will undoubtedly enable preventive intervention [2].

The Research Domain Criteria (RDoC) is constituted by a dimensional rather than a categorical system and easily enables to link different levels of molecule, circuit, behavior and symptom. From a neurodevelopmental perspective, it has been suggested that the RDoC may reveal the developmental trajectory at neuroanatomical level and sensitive periods of developmental period. Hence, it has been suggested that the integration of neurodevelpmental models and approaches to the RDoC in the etiology and pathogenesis of schizophrenia may enlighten the novel targets for intervention and specifying the timing of intervention. Moreover, it has been suggested that the reconceptualizing schizophrenia can be consistent with a novel bio-psychosocial-behavioral model dealing with complexity of the recognition and treatment [86]. The marked heterogeneity between patients with schizophrenia can be studied effectively with the RDoC rather than traditional diagnostic barriers [87,88]. The RDoC implies that targeted pharmacological treatment individualized to the patient, rather than measurement-based care is needed to understand and treat schizophrenia in terms of multi-dimensional natures in the DSM-5 [89].

## 5. A Novel Bio-Psychosocial-Behavioral Treatment Model in Schizophrenia

From the viewpoint of neurodevelopment, there may be stages of schizophrenia [84]. Using not only the clarification of subtle clinical features but also the advent of biomarkers and new cognitive tools, it may be possible to detect earlier stages of risk and prodrome [90]. Focused on cognitive and/or negative symptoms, Insel [84] has defined the stages of schizophrenia as follows: Stage I is characterized by genetic vulnerability and environmental exposure, possibility of being diagnosed with genetic sequence and family history, and little to no cognitive deficit. Stage II is characterized by the features of cognitive, behavioral and social deterioration, help seeking, being diagnosed through cognitive assessment, neuroimaging and the Structured Interview for Prodromal Syndrome (SIPS). Interventions at this stage could include cognitive training, treatment with polysaturated fatty acids, and social support. Stage III is characterized by the features of abnormal thought and behavior and relapsing-remitting course, as revealed through clinical interview, the loss of insight into one’s condition, being intervened with medications and psychosocial interventions. Lastly, stage IV is marked by continued deterioration, unemployment and homelessness, medical complications and incarceration. Interventions are medication, psychosocial interventions and rehabilitation services.

In accordance with reconceptualizing schizophrenia in terms of the neurodevelopment and neural circuitry perspective, defining a novel bio-psychosocial-behavioral treatment model for schizophrenia is needed to be consistent with staging care, personalized care, preventive approaches, reducing the cognitive deficits, integration of care and reducing stigma, which have been proposed as a vision for schizophrenia in 2030 [2,91].

## 6. Conclusions

In terms of the etiology and pathogenesis of schizophrenia, the current treatment target is focused on the dopamine, which is mainly associated with positive symptoms. However, cognitive and negative symptoms rather than positive symptoms have been more closely associated with functional recovery or remission in patients with schizophrenia. Targeting non-dopaminergic mechanisms including glutamate and GABA systems, focusing on the imbalance of excitatory-inhibitory signals caused by disturbances during early development. Hence, a novel bio-psychosocial-behavioral treatment model in schizophrenia is needed to target non-dopaminergic mechanisms rather than dopamine mechanism and be consistent with staging care, personalized care, preventative care, reducing cognitive deficits, and integrating psychiatric care.
